# Reconstitution of EPA and DHA biosynthesis in *Arabidopsis*: Iterative metabolic engineering for the synthesis of *n*−3 LC-PUFAs in transgenic plants

**DOI:** 10.1016/j.ymben.2013.03.001

**Published:** 2013-05

**Authors:** Noemi Ruiz-Lopez, Richard P. Haslam, Sarah L. Usher, Johnathan A. Napier, Olga Sayanova

**Affiliations:** Department of Biological Chemistry, Rothamsted Research, Harpenden, Herts AL5 2JQ, UK

**Keywords:** ALA, α-Linolenic acid, ARA, Arachidonic acid, DAG, Diacylglycerol, DHA, Docosahexaenoic acid, DPA, Docosapentaenoic acid, EPA, Eicosapentaenoic acid, GLA, γ-Linolenic acid, LA, Linoleic acid, LC-PUFA, Long chain polyunsaturated fatty acid, PC, Phosphatidylcholine, PE, Phosphatidylethanolamine, PI, Phosphatidylinositol, PS, Phosphatidylserine, SDA, Stearidonic acid, TAG, Triacylglycerol, Desaturase, Elongase, Nutritional enhancement, Omega-3 long chain polyunsaturated fatty acids, Transgenic plants

## Abstract

An iterative approach to optimising the accumulation of non-native long chain polyunsaturated fatty acids in transgenic plants was undertaken in *Arabidopsis thaliana*. The contribution of a number of different transgene enzyme activities was systematically determined, as was the contribution of endogenous fatty acid metabolism. Successive iterations were informed by lipidomic analysis of neutral, polar and acyl-CoA pools. This approach allowed for a four-fold improvement on levels previously reported for the accumulation of eicosapentaenoic acid in *Arabidopsis* seeds and also facilitated the successful engineering of the high value polyunsaturated fatty acid docosahexaenoic acid to 10-fold higher levels. Our studies identify the minimal gene set required to direct the efficient synthesis of these fatty acids in transgenic seed oil.

## Highlights

► Successful iteration of EPA and DHA accumulation in transgenic plants. ► Systematic identification of optimal genes for accumulation of DHA. ► Demonstration of the utility of lipidomic-driven plant metabolic engineering.

## 1 Introduction

Alternative sources of *n*−3 (also called omega-3) long chain polyunsaturated fatty acids (LC-PUFAs) have received considerable interest in recent years, based on the reduced availability of primary stocks (fish oils) and the clear evidence of health benefits from a diet that contains these fatty acids (Cressey, 2009; Saravanan et al., 2010). One approach to the sustainable supply of LC-PUFAs is the metabolic engineering of transgenic plants with the capacity to synthesise *n*−3 LC-PUFAs, since the global requirements for these fatty acids far exceeds what could be produced by algal or (recombinant) microbial fermentation (Domergue et al., 2005a). The “scalability” of agriculture-based production systems, combined with low input costs, make the development of a transgenic plant platform for the terrestrial synthesis of *n*−3 LC-PUFAs very appealing, although realising this goal has proved technically challenging to date (Sayanova and Napier, 2004; Robert, 2006; Dyer et al., 2008). This is due, in part, to the biochemical nuances of the *n*−3 LC-PUFA biosynthetic pathway as exemplified by the “substrate-dichotomy” bottleneck which exists between the phospholipid-dependent desaturases and the acyl-CoA-dependent elongases (Abbadi et al., 2004; Napier et al., 2004). Moreover, since higher plants have no endogenous capacity to synthesise these fatty acids, reconstruction (introduction) of the biosynthetic pathway demands the addition of multiple genes (for both primary synthesis and to direct the flux of substrate and biosynthetic intermediates towards final compartmentalisation in triacylglycerol) requiring co-ordinated tissue-specific expression in the developing seeds of a transgenic host (Cheng et al., 2010).

**Fig. 1:**
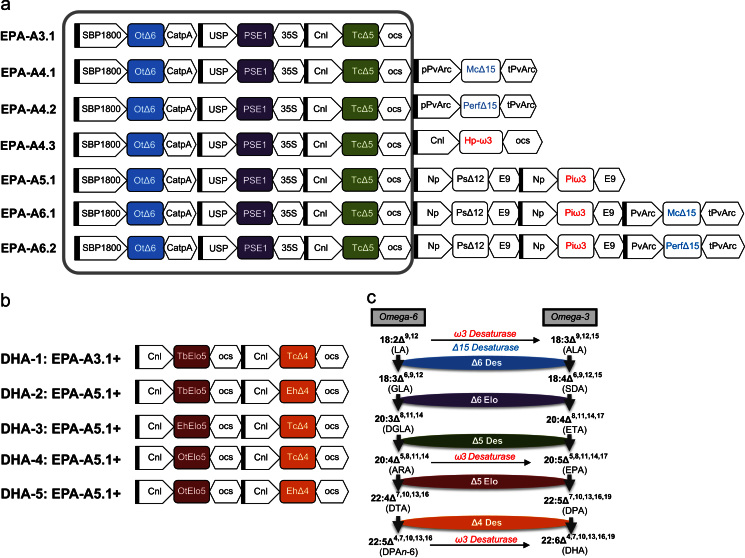
Representation of the constructs used for Arabidopsis transformation. (A) EPA contructs; (B) DHA constructs. Cnl=conlinin 1promoter for the gene encoding the flax 2S storage protein conlinin; USP=promoter region of the unknown seed protein of *V. faba*; SBP1800=the sucrose binding protein 1800 promoter; NP=napin; OtΔ6, a Δ6-desaturase from *O. taurii*; TcΔ5- a Δ5-desaturase from *Thraustochytrium* sp.; Pi ω3 and Hpω3—ω-3 desaturases from *P. infestans and H. parasitica*, respectively; PsΔ12- a Δ12-desaturases from *P. sojae*; PerfΔ15 and McΔ15–Δ15-desaturases from *P. fruticosa* and *M. chthonoplastes*, respectively; EhΔ4and Tc4- Δ4-desaturases from *E. huxleyi and Thraustochytrium sp.*; PSE1, a Δ6-elongase from *P. patens*, OtElo5- Δ5-elongase from *O. tauri* TbElo5, aΔ5-elongase from *T. brucei* and EhElo5, a bifunctional Δ5/Δ6-elongase from *E. huxleyi*; OCS, 35S, E9, PvARC and CatpA—represent terminators. The biosynthetic pathway for LC-PUFAs in shown schematically in C, with the different enzyme activies shown in different colours (also reflected in (A) and (B)). (For interpretation of the references to color in this figure legend, the reader is referred to the web version of this article.)

It is for this reason that the reconstruction of the *n*−3 LC-PUFA pathway represents the leading edge of metabolic engineering in transgenic plants—currently up to ten different genes have been stably introduced to different host species (Wu et al., 2005; Ruiz-Lopez et al., 2012). However, the accumulation of the target fatty acids such as eicosapentaenoic acid (20:5*n*−3; abbreviated to EPA) and especially docosahexaenoic acid (22:6*n*−3; abbreviated to DHA) have often proved disappointing, despite many attempts to optimise their synthesis (Venegas-Calerón et al., 2010). On one level, this is perhaps unsurprising, since the genes assembled to direct the synthesis of EPA and DHA (represented schematically by the pathway in Fig. 1C) are derived from a diverse set of *n*−3 LC-PUFA accumulating organisms, predominantly marine microbes such as diatoms and microalgae which form the base of the aquatic food-web. Early attempts to engineer the synthesis of EPA initially resulted in very low levels (<1% of total) of this fatty acid in seeds of transgenic linseed (Abbadi et al., 2004), but subsequent iterations increased the levels by at least 10-fold (though also increasing the undesired accumulation of biosynthetic intermediates) (Wu et al., 2005; Cheng et al., 2010). In both cases, these increases were achieved through the presence of additional enzyme activities, beyond the three primary biosynthetic activities used initially (Abbadi et al., 2004)—for recent consideration of these and other relevant studies, please consult these reviews (Ruiz-Lopez et al., 2012; Venegas-Calerón et al., 2010). Certainly, the predictive manipulation of plant seed oil compositions remains still in its infancy.

One significant advance in elevating the accumulation of LC-PUFAs in transgenic plants is the use of acyl-CoA-dependent desaturases, which bypass the above-mentioned substrate-dichotomy bottleneck. Elegant studies from Heinz and colleagues demonstrated the critical difference between phospholipid-dependent- and acyl-CoA-dependent desaturases in the heterologous reconstitution of LC-PUFA biosynthesis in yeast (Domergue et al., 2003) and these same researchers subsequently identified an acyl-CoA-dependent Δ6-desaturase from the picoalga *Ostreococcus tauri* which showed high activity in yeast towards LA-CoA and ALA-CoA (Domergue et al., 2005b). We and others have hypothesised that the use of acyl-CoA-dependent desaturases should result in increased synthesis and accumulation of EPA (Kajikawa et al., 2004; Graham et al., 2007; Petrie et al., 2010), although until recently, evidence for this has been lacking (Robert et al., 2005, Hoffmann et al., 2008). However, we have now demonstrated the benefits of using the *O. tauri* acyl-CoA dependent Δ6-desaturase, not only to elevate the accumulation of EPA, but also to avoid the accumulation of unwanted C18 biosynthetic intermediates (such as GLA and SDA), which are often associated with the expression of phospholipid-dependent Δ6-desaturases (Ruiz-Lopez et al., 2012; Sayanova et al., 2011; cf. Abbadi et al., 2004).

In this study, we build on those earlier observations to further optimise the accumulation of EPA, exclusively via the acyl-CoA-dependent desaturase pathway. Specifically, we wished to determine the contribution of both transgene-derived and endogenous activities on the accumulation of this target fatty acid. In addition, having generated a significant level of EPA in transgenic seeds, we systematically evaluated a number of different gene combinations to direct the synthesis of DHA. Using *Arabidopsis* as a well-established model system for the metabolic engineering of the *n*−3 LC-PUFA pathway (Qi et al., 2004; Robert et al., 2005; Hoffmann et al., 2008; Sayanova et al., 2011), we have successfully iterated the accumulation of DHA to 10-fold that of the levels previously reported in this species.

## 2 Material and methods

### 2.1 Plant material and growth conditions

*Arabidopsis thaliana*, Columbia (Col-0) ecotype, was grown for analyses in a controlled environment chamber at 23 °C day/18 °C night, 50–60% humidity, and kept on a 16-h, 250 μmol m^−2^ s^−1^, photoperiod (long day).

#### 2.1.1 Generation of transgenic plants

Transgenic *Arabidopsis* lines were generated as previously described (Sayanova et al., 2006). Copy number of the T-DNA insertion was not determined. In all cases, no phenotypic alteration of the plants were observed on modification of the seed oil composition.

### 2.2 Vector construction

This was essentially as described in Ruiz-Lopez et al. (2012). All open reading frames for desaturases and elongases were resynthesized (GenScript Corporation, NJ) and codon-optimised for expression in *Arabidopsis*.

#### 2.2.1 EPA constructs

Seven constructs containing from 3- to 7-gene cassettes were built using the Gateway^®^ recombination system (Invitrogen) as described in Ruiz-Lopez et al. (2012). Respective genes were inserted as *Nco*I/*Pac*I or Asc/Pac fragments into the promoter/terminator cassettes and then moved into pENTRY vectors. The core construct containing minimal set of genes required for the production of EPA (designated A3.1) comprised of the three expression cassettes, including (1) the sucrose binding protein SBP1800 promoter, OtΔ6, a Δ6-desaturase gene from *O. tauri* (Domergue et al., 2005b) and CatpA, terminator; (2) USP1 promoter (Bäumlein et al., 1991), PSE1, a Δ6 fatty acid elongase from *Physcomitrella patens* (Zank et al., 2002) and CaMV35S terminator; (3) Cnl, a conlinin1 promoter (Truksa et al., 2003), TcΔ5, a Δ5-desaturase from *Thraustochytrium* sp. (Qiu et al., 2001) and OCS, a terminator region of OCS, octopin synthase gene of *Agrobacterium tumefaciens* (Fig. 1A).

The 4-gene-construct, designated A4.1, A4.2 and A4.3 were designed by adding three different ω3 desaturase genes to the core construct A3.1. A4.1 and A4.2 contained additional Fatty Acid Desaturase3 genes: McΔ15, a Δ15 fatty acid desaturase gene from *Microcoleus chthonoplastes* (Senger and Bauer, 2010) was inserted into A3.1 to produce A4.1 and PerfΔ15, a Δ15 fatty acid desaturase gene from *Perilla fruticosa* (Chung et al., 1999) was inserted into A3.1 to produce A4.2. Both genes were under control of PvArc promoter and linked to a PvArc terminator region. Construct A4.3 contained a novel C20-specific ω3 desaturase Hp-ω3 gene from *Hyaloperonospora parasitica* (Senger and Bauer, 2010) cloned under the control of the conlinin-1 promoter and linked to OCS terminator.

A similar approach was used to build the five gene construct A5.1. Two gene cassettes consisting of PsΔ12, a Δ12-desaturase gene from *Phytophthora sojae* and Piω3, a ω3 desaturase gene from *Phytophthora infestans* (Wu et al., 2005) flanked by the *Brassica napus* napin promoter and E9 terminator regions were added to A3.1 to produce A5.1 (Fig. 1A).The same approach was used to design the six-gene constructs (Fig. 1A) by inserting FAD3 genes into A5.1: A6.1 included McΔ15 and A6.2 included PerfΔ15 genes.

#### 2.2.2 DHA constructs

For the five-gene DHA-1 construct two more gene cassettes containing TbElo5, a *Trypanosoma brucei* Δ5 fatty acid elongase gene (Livore et al., 2007) and TcΔ4, a Δ4 fatty acid desaturase gene from *Thraustochytrium* sp. (Qiu et al., 2001), both under control of conlinin promoters were cloned into the triple gene construct A3.1 (Fig. 1B).

To build the seven-gene DHA-2 construct, two gene cassettes, containing TbElo5, and EhΔ4, a Δ4-desaturase gene from *Emiliania huxleyi* (Sayanova et al., 2011), both flanked by conlinin promoters and OCS terminators were added to the A5.1 construct.

For the design of the seven-gene constructs DHA-3, DHA-4 and DHA-5, asimilar approach was used to add Δ5 fatty acid elongase and Δ4 fatty acid desaturase genes flanked by conlinin promoters and OCS terminators to five-gene A5.1 construct: DHA-3 contained two additional gene cassettes expressing EhElo5/Elo6, a bifunctional Δ5/Δ6-elongase from *E. huxleyi* (Sayanova et al., 2011) and TcΔ4-desaturase; DHA-4 included OtElo5, an *O. tauri* Δ5 fatty acid elongase gene (Wu et al., 2005) and the TcΔ4desaturase gene and DHA-5 construct contained the OtElo5 and EhΔ4 sequences (Fig. 1B).

### 2.3 Fatty-acid analysis

Fatty acids were extracted and methylated as described (Sayanova et al., 1997,2003). Methyl ester derivatives of total fatty acids extracted were analysed by GC and GC–MS. Data presented as representative numbers derived from replicated analysis.

### 2.4 Acyl-CoA profiling

cTwenty-milligrams of developing (15 days after flowering) seed material were collected, frozen in liquid nitrogen and extracted after Larson and Graham (2001), for reverse-phase LC with either quantitative analysis of fluorescent acyl-etheno-CoA derivatives or with electrospray ionization tandem mass spectrometry (multi reaction monitoring) in positive ion mode. For the analysis of etheno-CoA derivatives HPLC (Agilent 1200LC system; Phenomenex LUNA 150 2 mm C18(2) column) was performed using the methodology and gradient conditions described previously (Larson and Graham, 2001); whilst LC–MS/MS +MRM analysis (AB4000 QTRAP) followed the methods described by Haynes et al., 2008 (Agilent 1200LC system; Gemini C18 column, 2 mm inner diameter, 150 mm with 5 mm particles). For the purpose of identification and calibration, standard acyl-CoA esters with acyl chain lengths from C14 to C20 were purchased from Sigma as free acids or lithium salts.

### 2.5 Lipid extraction and separation

Three hundred milligrams of seeds were heated for 10 min at 95 °C in 1 mL of isopropanol and homogenized using a mortar and pestle. The homogenate was centrifuged, supernatant collected, and the pellet re-extracted with isopropanol: chloroform (1:1, v/v). Both extracts were pooled, evaporated, and dissolved in chloroform: acetic acid (100:1, v/v). The lipid extract was loaded on a Sep-pak column and pre-fractionated into neutral lipids, glycolipids, and phospholipids adding chloroform: acetic acid (100:1, v/v), acetone: acetic acid (100:1), and methanol, respectively. These fractions were further resolved on thin-layer chromatography silica gel plates, thickness 0.25 mm. Neutral lipids were developed with hexane: ethyl ether: formic acid (75:25:1, by volume), and polar lipids with chloroform: methanol: ammonia: water (70:30:4:1, by volume). The individual lipid classes were identified under UV light after a primuline spray (0.05% [w/v] in acetone: water, 80:20, v/v), scraped from the plate, and used directly for methylation or extracted for further analysis. Lidipomic analysis was carried out as previously described (Ruiz-Lopez et al., 2012).

## 3 Results and discussion

### 3.1 Enhancing EPA biosynthesis in transgenic *Arabidopsis*

To further optimize the production of EPA in oilseed crops using the more efficient acyl-CoA-dependent biosynthetic pathway, we describe a stepwise engineering approach to generate a range of different constructs carrying three to six genes (with each gene under the control of a seed-specific promoter). These constructs were introduced into *Arabidopsis* plants via floral dip transformation and mature seeds from kanamycin-resistant T2 plants were then analysed by GC–FID for total fatty acid composition. No specific attempt was made to isolate homozygous lines from subsequent kanamycin resistant progeny.

**Fig. 2:**
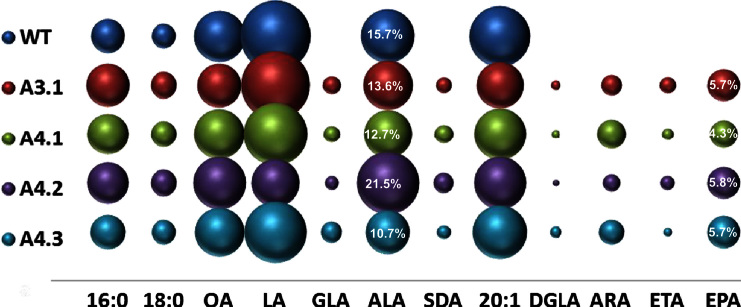
Graphical representation of the accumulation of fatty acids in transgenic Arabidopsis plants containing constructs A3.1-A4.3 The mean mol% of individual fatty acids (*Y*-axis) is directly proportional to the size of the sphere. Different constructs are shown on the *X* axis as indicated. For reference, the mol% accumulation of the target fatty acid EPA is given numerically within the relevant sphere, as is the mol% for the endogenous fatty acid ALA. A full description of the fatty acid composition of these lines is given in Supplementary Table 1. Lines are T2 generation.

The first construct (designated A3.1), contained the minimal three genes required for the synthesis of *n*−3/*n*−6 C20 LC-PUFAs (e.g. EPA and ARA) from endogenous C18 substrates (represented schematically in Fig. 1A and C). Specifically, a Δ6-desaturase gene from *O. tauri* (OtΔ6; Domergue et al., 2005b), a Δ6 fatty acid elongase gene from *P. patens* (PSE1; Zank et al., 2002) and a Δ5-desaturase gene from *Thraustochytrium* sp. (TcΔ5; Qiu et al., 2001) were individually cloned behind seed-specific promoters and then combined into one single T-DNA transformation vector as previously described (Ruiz-Lopez et al., 2012). Analysis of total fatty acid methyl-esters (FAMEs) from the seeds of 20 individual transgenic *Arabidopsis* T2 lines indicated that plants expressing the A3.1construct accumulated significant levels of EPA (Fig. 2; see also Supplementary Table 1 for full numerical presentation these data).The mean level of EPA found in *Arabidopsis* seeds was 5.7%, which is higher than we and others have observed in *Arabidopsis*. In agreement with previous results obtained with the expression of acyl-CoA Δ6-desaturases in transgenic plants (Hoffmann et al., 2008; Petrie et al., 2010 Sayanova et al., 2011; Ruiz-Lopez et al., 2012) only a minor accumulation of C18 Δ6-desaturated fatty acids was observed. The mean levels of GLA and SDA in T2 seeds ranged from 0.4% to 3.9% (average 1.7%) and 0.4% to 2.1% (average 1.2%) of total fatty acids, respectively (Fig. 2, Supp. Table 1). The *n*−6 LC-PUFA ARA accumulated from 0.4 to 6.4% (average 2.2%). Interestingly, seeds which contained higher levels of EPA displayed reduced levels of ARA, implying variation in the endogenous channelling of fatty acids into either pathway. Overall levels of newly synthesised *n*−3 fatty acids were much higher than that of *n*−6 acids, with an *n*−3/*n*−6 ratio 3:1 in the best lines of the basic A3.1 construct.

To determine if the presence of ω3-desaturases could increase the levels of *n*−3 fatty acids, three different activities were added independently to the A3.1 construct—two genes encoding FAD3-like Δ15-desaturases and one gene encoding a C20-specific ω3 desaturase. In these configurations, it was predicted that either increased ALA substrate would be generated (by FAD3) or *n*−6 products such as ARA would be reduced (by the C20 ω3-desaturase), with both activities leading to increased EPA. These new constructs are represented in Fig. 1. The first two constructs, containing FAD3-like sequences were A4.1, containing the Δ15-desaturase from the cyanobacterium *M. chthonoplastes* (McΔ15) plus OtΔ6, PSE1 and TcΔ5; and A4.2, containing the Δ15-desaturase from the higher plant *P. fruticosa* (PerfΔ15) gene plus OtΔ6, PSE1 and TcΔ5. Fatty acid analysis of T2 seeds from 20 representatives for each construct revealed that the most abundant non-native species was EPA. In transgenic seeds of A4.1 and A4.2 lines the highest levels of EPA found were 5.3% and 7.4%, with the average values of 4.3% and 5.8% of total seed fatty acids, respectively (Fig. 2, Supp. Table 1). In agreement with previous results, low levels of the intermediate fatty acids GLA and SDA were obtained. The addition of the McΔ15 FAD3 ω3-desaturase to the core A3.1 construct had no positive effect on the levels of ALA or EPA, although mean levels of LA were decreased (from 27.9% in WT to 22.4% in transgenic lines). Counter-intuitively, the levels of ARA in A4.1 were increased with the addition of the McΔ15 desaturase (from 2.2% to 4.6%). Although the McΔ15 FAD3 ω3-desaturase is cyanobacterial in origin, and might not be expected to contribute significantly to extra-plastidial lipid modification, previous studies have confirmed that this enzyme has activity in eukaryotic cells (Senger and Bauer, 2010) in line with our observations of perturbations to acyl composition in our transgenic seeds (Supp. Table 1). In contrast, expression of the PerfΔ15FAD3 microsomal desaturase in A4.2 lines had a clear effect on both endogenous and non-native fatty acids. In particular, T2 seeds of A4.2 contained a substantially increased ALA content (from an average of 15.7% in WT to 21.5% in transgenic seeds) (Supp. Table 1). More strikingly, the LA content was decreased dramatically from the average levels of LA of 27.9% in WT to 13.0% in transgenic lines (Supp. Table 1). Despite this radical reconfiguration of endogenous fatty acids which serve as substrates for the transgene-encoded LC-PUFA pathway, no significant increase in the levels of EPA was detected in A4.2 (5.8% total seed fatty acids versus 5.7% total seed fatty acids for A3.1) (Fig. 2, Supp. Table 1). This is in spite of the increased levels of ALA (1.7-fold greater in A4.2 versus A3.1)—these data confirm that there is no obvious substrate-product relationship between C18 and C20 *n*−3 total fatty acids for these constructs. In contrast, average levels of the C20 *n*−6 ARA were reduced in A4.2, down to a mean of 1.5% (range 0.8–2.5%), compared to an average value of 4.6% (range 4.0–5.8%) in A4.1 and 2.2% in A3.1 (Supp. Table 1). This indicates that PerfΔ15 has a major effect on shifting the flux from the *n*−6 to the *n*−3 pathway in transgenic *Arabidopsis*, but that this does not significantly increase EPA accumulation in transgenic seeds. However, the presence of this activity does result in both a reduction of total *n*−6 fatty acids, and increased levels of ALA, resulting in a more favourable *n*−3/*n*−6 ratio of 1.9 (compared with 0.6 in A4.1).

Recently, we isolated a new C20-specific ω3-desaturase from *H. parasitica* (designated Hp-ω3). In yeast, this desaturase effectively converted *n*−6 ARA to *n*−3 EPA (Supp. Table 2). To determine if Hp-ω3 could efficiently convert ARA to EPA in transgenic plants we added this gene to the core A3.1 configuration to generate the four-gene construct A4.3. Fatty acid analysis of seeds from T2 transgenic plants revealed that the inclusion of the Hp-ω3 C20 ω3-specific desaturase did not produce a meaningful decrease in ARA (Supp. Table 1). Interestingly, the LA levels in A4.3 plants were decreased (27.9% in WT and 21.1% inA4.3), indicating that Hp-ω3 also has FAD3-like C18 Δ15-desaturase activity in the metabolic context present in transgenic *Arabidopsis*. Unexpectedly, levels of ALA also went down from 15.7% in WT to 10.7%, although there was no substantial increase in the average levels of EPA generated by this construct (though the range observed was greater, with maximal levels of EPA in excess of 10% in two individual lines) (Supp. Table 1). Similarly, there was no obvious correlation between the levels of LA, ALA and EPA. Based on these data, the benefits of using ω3-desaturases (either C18 or C20-specific) to boost the accumulation of EPA appear to be limited, at least in *Arabidopsis*. Previous studies by Wu et al. (2005) on the accumulation of EPA in transgenic *Brassica juncea* revealed a significant increase in this fatty acid resulting from the co-expression of a C20 ω3-desaturase from *P. infestans* (from 1.4% to 8.1% EPA, with a concomitant decrease in the levels of ARA—from 13.7% to 5.4%). Similarly, Cheng et al. (2010) observed a clear contribution of C20 ω3-desaturation to elevated levels of EPA in transgenic *Brassica carinata*, with levels rising from 2.3% in a core 3-gene construct to 4.2% EPA with the addition of the CpDesX ω3-desaturase from *Claviceps purpurea*, and further increasing to 9.7% EPA on the additional co-expression of an ω3-desaturase from *Pythium irregulare*. More recently, we observed some moderate enhancement of EPA accumulation in *Arabidopsis* seeds with the co-expression of the same *P. infestans* ω3-desaturase described above (Ruiz-Lopez et al., 2012; Wu et al., 2005). Collectively, all these studies indicate that whilst ω3-desaturases can contribute to increasing the transgenic synthesis and accumulation *n*−3 LC-PUFAs such as EPA, this effect is likely to be modulated by the configuration of endogenous seed lipid metabolism of the host plant. Given that this varies from species to species, as witnessed by the variation in seed oil composition in plants, this highlights the need for a better understanding of the biochemical context into which transgenic metabolic pathways are inserted. An additional factor which may influence the efficacy of ω3-desaturation in converting non-native *n*−6 LC-PUFAs to *n*−3 forms is whether or not the transgenic pathway is operating via the acyl-CoA pool or phospholipids—in the example of Wu et al. (2005), this was utilising a phospholipid-dependent Δ6-desaturase, whereas in this study we used the *O. tauri* acyl-CoA-dependent Δ6-desaturase. Thus, ω3-desaturation (likely utilising phospholipid substrates) may be a less useful “skew” in a acyl-CoA biosynthetic pathway.

### 3.2 Investigating the effect of additional activities on EPA production in transgenic *Arabidopsis*

**Fig. 3:**
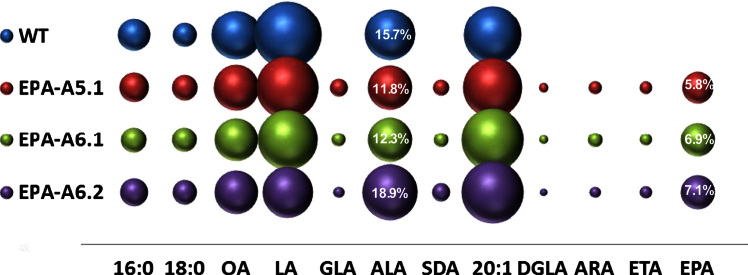
Graphical representation of the accumulation of fatty acids in transgenic Arabidopsis plants containing constructs A5.1-A6.2. The mean mol% of individual fatty acids (*Y*-axis) is directly proportional to the size of the sphere. Different constructs are shown on the *X* axis as indicated. For reference, the mol% accumulation of the target fatty acid EPA is given numerically within the relevant sphere, as is the mol% for the endogenous fatty acid ALA. A full description of the fatty acid composition of these lines is given in Supplementary Table 3. Lines are T2 generation.

In a second iteration designed to increase the transgenic accumulation of EPA, additional enzyme activities were added to augment the activities described above. Thus, a five-gene construct designated A5.1 was generated by combining the genes present in A-3.1 with a Δ12-desaturase gene from *P. sojae* (PsΔ12) to enhance the levels of LA-CoA (as substrate for the OtΔ6 enzyme) and an ω3-desaturase from *P. infestans* (Pi-ω3; Wu et al., 2005; Ruiz-Lopez et al., 2012) to increase the conversion of ARA to EPA (Fig. 1A and C). Forty T2 transgenic *Arabidopsis* lines for A5.1 were identified on the basis of the presence of non-native fatty acids, as detected by GC-FID analysis of total seed fatty acids. As expected and previously reported (Ruiz-Lopez et al., 2012), only a minor accumulation of Δ6-desaturated fatty acids was observed (average values of 1.9% GLA and 1.4% SDA) in T2 seeds (Fig. 3; Supp. Table 3). The amount of ARA present in the seeds of A5.1 was low, ranging from 0.4% to 1.8% (average1.0%), whilst EPA accumulated to an average level of 5.8% (with a range of 4.3% to 10.5% total seed fatty acids) (Fig. 3). The levels of LA and ALA in A5.1 transgenic seeds showed some minor reduction when compared with WT (Supp. Table 3). Collectively these data confirmed our recent observations on the limited impact of these two desaturases (PsΔ12, Pi-ω3) on enhancing the accumulation of EPA (Ruiz-Lopez et al., 2012).

In view of the these results, we performed a final iteration, in which genes encoding Δ15-desaturases were inserted into A5.1 to produce two different six-gene configurations. The addition of the cyanobacterial McΔ15 desaturase (to generate A6.1) or the addition of the higher plant FAD3 desaturase PerfΔ15 (to generate A6.2) yielded two constructs which were then introduced in *Arabidopsis* as described above. GC-FID analysis of fatty acids from T2 seeds, encompassing 34 representatives for each construct, demonstrated that EPA was the most abundant non-native FA species. The highest measured levels of EPA observed were 12.4% and 11.7% (average 7.1% and 6.9%) for constructs A6.2 and A6.1, respectively (Fig. 3; Supp. Table 3). Only minor accumulation of C18 Δ6-desaturated fatty acids was observed in transgenic seeds expressing either construct, with an averaged value of less than 3.7% for GLA and less than 3.9% for SDA. The content of ARA was also low (ranging from 0.7% to 2.8% in A6.1 and from 0.3% to 1.4% in A6.2; Supp. Table 3).

The inclusion of the McΔ15 FAD3 ω3-desaturase in construct A6.1 had no obvious effect on the levels of ALA, although levels of LA showed some decrease (from 27.9% in WT to 22.0% in transgenic lines). However, EPA levels showed a notable increase (from 5.8% in A5.1 line to 6.9% in A6.1 line). In the case of construct A6.2, the co-expression of the PerfΔ15 C18 ω3-desaturase again resulted in a major decrease in LA levels (from an average of 27.9% in WT to 15.5% in transgenic plants) and in a substantial increase of ALA levels (from an average values of 15.7% in WT to 18.9%) (Supp. Table 3). The mean level of EPA for A6.2 (7.1%) was the highest we have observed with any of our current iterations, and compares very favourably (at least double) with previous reports of others (Robert et al., 2005; Hoffmann et al., 2008). In these constructs we observed a statistically significant correlation of the increased levels of ALA and high levels of EPA: in some high EPA-plants the levels of EPA were ∼12%, levels of ALA ∼20%, while levels of LA were reduced to 11% (Supp. Table 3).

Collectively, these data indicate that altering many lipid biosynthetic activities have limited impact on the seed oil composition of *Arabidopsis*. The most dramatic change resulted from the co-expression of the higher plant FAD3 activity from *P. fructans*, generating a radical shift in the levels and ratio of LA and ALA. Our data mirrored the previous observations of Puttick et al. (2009) who over-expressed FAD3 in the seeds of *Arabidopsis*, resulting in a dramatic 2.5-fold increase in ALA and concomitant reduction in LA levels. Our data also implies that some key nodes in fatty acid metabolism are under strong homeostatic control.

### 3.3 Expressing EPA constructs in the *fae1-1 Arabidopsis* mutant background

In order to evaluate the impact of endogenous accumulation of C20+ monounsaturated fatty acids on the transgenic accumulation of *n*−3 LC-PUFAs, constructs A5.1 and A6.1 were introduced into the *fae1-1* background of *Arabidopsis*—this mutant lacks the *FAE1* condensing enzyme activity and is defective in the elongation of oleic acid (Lemieux et al., 1990). Consequently, this mutant has elevated levels of oleic acid and LA but only trace amounts of 20:1 *n*−9 eicosanoic acid. Previous studies by Cheng et al. (2010) indicated that an analogous mutant background (zero-erucic) in *B. carinata* had a significant enhancement on the transgenic accumulation of EPA, increasing from 9% to 20% of total seed oil. Fatty acid analysis of T2 seeds from 40 individual *Arabidopsis* lines expressing each construct revealed that the EPA content in *fae1-1* transgenic seeds was only slightly higher than that in corresponding WT *Arabidopsis* transgenics (8.3% in A5.1 and 7.3% in A6.1 lines; Fig. 43, Supp. Table 4). These data are in stark contrast to those of Cheng et al. (2010) and require some further consideration as to the basis of the difference between *Arabidopsis* and *B. carinata* as hosts for heterologous LC-PUFA biosynthesis. One scenario is that the synthesis of 22:1*n*−9 in *B. carinata* is configured in a significantly different manner compared to the synthesis of 20:1 *n*−9 in *Arabidopsis* though perhaps the most obvious difference between our present study and that of Cheng et al. is that they used a phospholipid-dependent Δ6-desaturase whereas we used an acyl-CoA-dependent form. However, based on the limited impact of the *fae1-1* mutation on EPA synthesis in *Arabidopsis*, it seems likely that competition between endogenous and transgene condensing enzymes for either reductant (NADH) or malonyl-CoA is not limiting in this species.

**Fig. 4:**
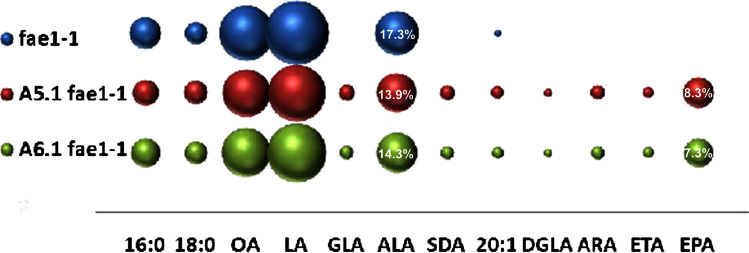
Graphical representation of the accumulation of fatty acids in transgenic Arabidopsis plants containing constructs A5.1 and A6.1 in the fae1-1 mutant background. The mean mol% of individual fatty acids (*Y*-axis) is directly proportional to the size of the sphere. Different constructs are shown on the *X* axis as indicated. For reference, the mol% accumulation of the target fatty acid EPA is given numerically within the relevant sphere, as is the mol% for the endogenous fatty acid ALA. A full description of the fatty acid composition of these lines is given in Supplementary Table 4. Lines are T2 generation.

**Fig. 5:**
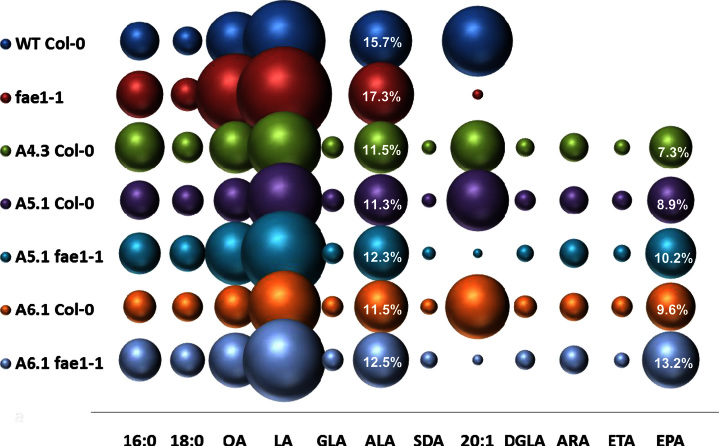
Graphical representation of the accumulation of fatty acids in transgenic Arabidopsis plants at T2 or T3 generation. The mean mol% of individual fatty acids (*Y*-axis) is directly proportional to the size of the sphere. Different constructs are shown on the *X* axis as indicated. For reference, the mol% accumulation of the target fatty acid EPA is given numerically within the relevant sphere, as is the mol% for the endogenous fatty acid ALA. A full description of the fatty acid composition of these lines is given in Supplementary Table 5.

**Fig. 6:**
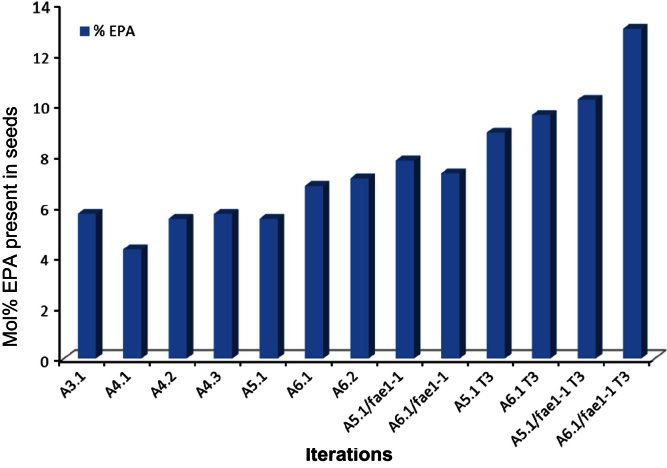
Increase in EPA across different iterations. Graph of mean EPA content (mol% of total seed fatty acids) observed in the different iterations described in this study.

It is also interesting to note the stability of the EPA trait derived from the expression of A5.1 and A6.1 cassettes in WT *Arabidopsis* and the *fae1-1* mutant. Accumulation of non-native fatty acids was monitored at the T3 generation derived from selected T2 plants (Supp. Tables 4 and 5; Figs. 4 and 5). EPA levels in T3 seeds increased in average from 5.5% to 8.9% in A5.1 plants (maximum yield 11.9%) and from 6.8% to 9.6% A6.1 plant (maximum yield 13%) (Supp. Table 5). EPA levels in the T3 seeds of transgenic *Arabidopsis* mutant *fae1-1* lines were slightly higher: 10.2% (max 12.2%) in A5.1/*fae1-1* plants and 13.2% (max 16.7%) in A6.1 plants (Supp. Table 5). As shown in Fig. 6, over the course of the iterations reported in this study, transgenic accumulation of EPA has more than doubled (from 5.7% to 13.2%), and representing a 4-fold increase in the highest levels previously reported for *Arabidopsis* (3.2%; Robert et al., 2005).

### 3.4 Reconstitution of DHA biosynthesis in *Arabidopsis*

**Fig. 7:**
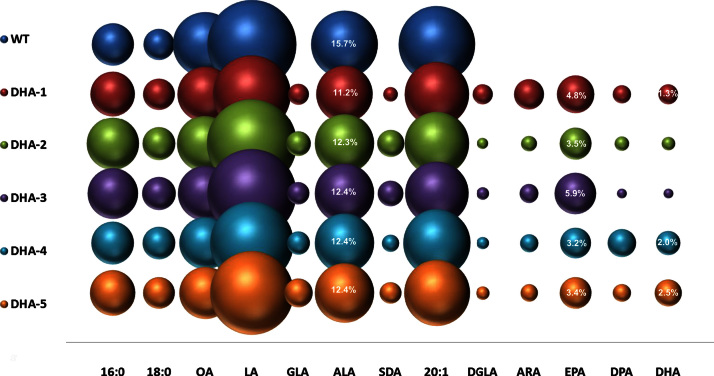
Graphical representation of the accumulation of DHA in transgenic Arabidopsis plants containing constructs DHA-1 to DHA-5. The mean mol% of individual fatty acids (*Y*-axis) is directly proportional to the size of the sphere. Different constructs are shown on the *X* axis as indicated. For reference, the mol% accumulation of the target fatty acids EPA and DHA are given numerically within the relevant spheres, as is the mol% for the endogenous fatty acid ALA. A full description of the fatty acid composition of these lines is given in Supplementary Table 6. Lines are T2 generation.

The identification of transgene combinations which successfully directed the accumulation of meaningful levels of EPA allowed us to elaborate expression cassettes for the introduction of the two additional activities required for DHA production i.e. a C20 Δ5-elongase and a Δ4 fatty acid desaturase. To create a minimal five-gene construct, the C20 Δ5 fatty acid elongase from *T. brucei* (TbElo5; Livore et al., 2007) and a Δ4 fatty acid desaturase gene from *Thraustochytrium* sp. (TcΔ4; Qiu et al., 2001) were added to A3.1 to create a construct designated DHA-1. We also used A5.1 as a more elaborate context for the evaluation of different combinations of Δ5 fatty acid elongase and Δ4 fatty acid desaturase activities, generating four different 7-gene DHA constructs. Thus, we added TbElo5 and EhΔ4, a Δ4-desaturase gene from *E. huxleyi* (Sayanova et al., 2011) to A5.1 to produce DHA-2; EhElo5, a bifunctional Δ5/Δ6-elongase gene from *E. huxleyi* (Sayanova et al., 2011) and TcΔ4 to produce DHA-3; OtElo5, an *O. tauri* Δ5 fatty acid elongase gene (Meyer et al., 2004) and TcΔ4 to produce DHA-4. Finally, we designed seven-gene DHA-5 construct containing OtElo5 and EhΔ4. All Δ5 fatty acid elongases and Δ4 fatty acid desaturase genes were flanked by the seed-specific conlinin promoter and OCS terminator (see Fig. 1B). Depending on which construct was introduced into *Arabidopsis*, varying mean levels of DHA were observed in total seed lipids (Fig. 7; Supp. Table 6); although all iterations generated significantly higher levels than those previously reported in *Arabidopsis* (0.3%, Robert et al., 2005). The lowest levels of DHA we observed in T2 transgenic seeds were with construct DHA-3 (containing the EhElo5 and TcΔ4 activities) yielding 0.2–0.8% of this fatty acid. This poor conversion of EPA to DHA was most likely due to limited activity of the bi-functional Δ5/Δ6-elongase from *E. huxleyi* in *Arabidopsis*. Expression of the Δ5 fatty acid elongase from *T. brucei*, TbElo5, in conjunction with the Δ4 fatty acid desaturase from *Thraustochytrium* sp. or the Δ4-fatty acid desaturase from *E. huxleyi* resulted in more significant amounts of DHA: 0.9–2.0% of total fatty acids in DHA-1 plants and 0.3% to 1.1% in DHA-2 plants. The highest levels of DHA were observed in transgenic seeds converting EPA in the presence of OtElo5, an *O. tauri*Δ5 fatty acid elongase used in conjunction with either TcΔ4 or EhΔ4 desaturases. The average yield of DHA in DHA-4 seeds was 2.0% of total fatty acids (with a range of 1.1% to 3.8%); whereas in DHA-5 seeds the mean was 2.5% (the highest value observed in individual line was 4.7%) (Supp. Table 6).

Very recently, Petrie et al. (2012) have described a single construct containing seven genes which successfully directed the seed-specific accumulation of DHA in transgenic *Arabidopsis*. This construct (pJP3416_GA7) yielded DHA in the range ∼3–6.5% in T2 and 3–9.5% DHA in T3 (Petrie et al., 2012). One individual event displayed significantly higher levels of DHA in T3 and T4 generations (14–15% of total seed fatty acids). It will be important to determine if such impressive levels are routinely achieved with this construct and also if they are transferable to other species. A metabolic explanation for the higher levels of DHA achieved by Petrie et al. (2012) is currently lacking, but indicates the basis for future studies.

### 3.5 Lipid analysis of transgenic *Arabidopsis* accumulating C22 *n*−3 LC-PUFAs

**Fig. 8:**
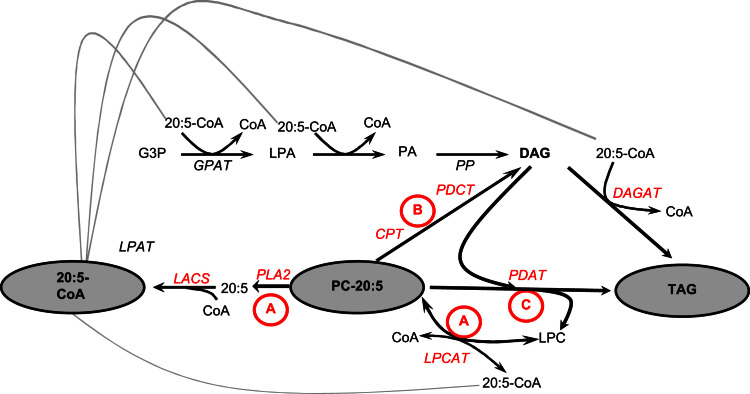
Schematic diagram of the main lipid classes and biochemical pathways involved in the channelling of fatty acids into TAG in developing seeds. The three primary routes for the incorporation of non-native LC-PUFAs such as EPA into TAG are shown (mechanisms A, B, and C). For mechanism A, EPA esterified to PC (i.e. site of synthesis) is under a constant dynamic exchange with the acyl-CoA pool in a process described as acyl-editing. Removal of EPA from PC can proceed by the reverse action of acyl-CoA:lysophosphatidylcholine acyltransferase (LPCAT) or the combined action of phospholipase A2 PLA_2_ and long-chain acyl-CoA synthetase, LACS. Once in the acyl-CoA pool, EPA-CoA and glycerol-3-phosphate (G3P) can be converted into TAG by the consecutive action of acyl-CoA:glycerol 3-phosphate acyltransferase (GPAT), acyl-CoA:lysophosphatidic acid acyltransferase (LPAT), phosphatidic acid phosphatase (PAP), and acyl-CoA:diacylglycerol acyltransferase (DGAT) in a series of reactions known as the Kennedy pathway. For mechanism B, the PC head group can be removed, producing a DAG molecule containing EPA at the sn-2 position. This reaction can proceed by four enzymatic mechanisms: phospholipase C, phospholipase D along with PAP, the reverse action of CDP-choline: diacylglycerol cholinephosphotransferase (CPT), or the recently identified phosphatidylcholine:diacyglycerol cholinephosphotransferase, (PDCT). The DAG produced by these mechanisms can then be utilized to produce TAG. For mechanism C, direct transfer of the sn-2 EPA of PC to the sn-3 of DAG produces TAG via a phospholipid:diacylglycerol acyltransferase (PDAT).

To better understand the endogenous metabolic constraints on the accumulation of non-native C22 *n*−3 LC-PUFAs, we carried out detailed lipidomic analysis of seeds containing different transgene combinations – this approach has previously been successfully used to identify bottlenecks in the accumulation of C20 fatty acids such as EPA (Ruiz-Lopez et al., 2012) – a schematic representation of the various pathways by which fatty acids can ultimately reach TAG is shown in Fig. 8. Analysis of the neutral lipid composition in mature seeds of WT, A5.1, A5.1/*fae1-1* and DHA-5 indicated that the highest levels (4.3%) of DHA accumulated in diacylglycerol (DAG), compared with 2.5% in triacylglycerol (TAG) (Table 1). This was also true for the accumulation of EPA, either for the DHA-synthesising configuration DHA-5 or the progenitor A5.1 construct for EPA alone. Conversely, the distribution of 20:1 *n*−9 was enriched in TAG as opposed to DAG, as previously observed (Bates and Browse, 2011).

Analysis of the polar lipid fractions confirmed our previously reported preferential accumulation of C20+ LC-PUFAs in phosphatidylethanolamine (PE) species (Ruiz-Lopez et al., 2012). For the DHA-synthesising line DHA-5, levels of DHA in PE were 9.5%, compared with 5.5% for phosphatidylcholine (PC) and 1.7% for the phosphatidylinositol (PI) and phosphatidylserine (PS) combined fraction. The levels of EPA in these same lines were equally enriched in PE (Table 2). For the EPA-synthesising lines, no significant difference was observed in the distribution of EPA between PE, PC and the PI+PS combined fraction, though it was noteworthy that the accumulation of EPA in the phospholipids of the *fae1-1* background was noticeably lower—the explanation for this is not obvious. Equally of note was the accumulation of GLA in the phospholipid fractions of all three configurations (Table 2); this was surprising given the presence of the *O. tauri* acyl-CoA-dependent Δ6-desaturase, which is known to bypass the phospholipid pool as part of the synthesis of GLA. However, there was no obvious enrichment of EPA and/or DHA in DAG versus PC, as previously observed with phospholipid dependent Δ6-desaturases in *Arabidopsis* (Ruiz-Lopez et al., 2012), commensurate with the presence of the *O. tauri* acyl-CoA-dependent activity. Positional analysis of PC to determine the location of DHA indicated a 3-fold enrichment at the sn-2 position compared with the sn-1 position (Table 3). This was evident for EPA, SDA and GLA, whereas many endogenous fatty acids (such as 18:1, ALA, 20:1) were biased towards the sn-1 position of PC (Table 3).

**Fig. 9:**
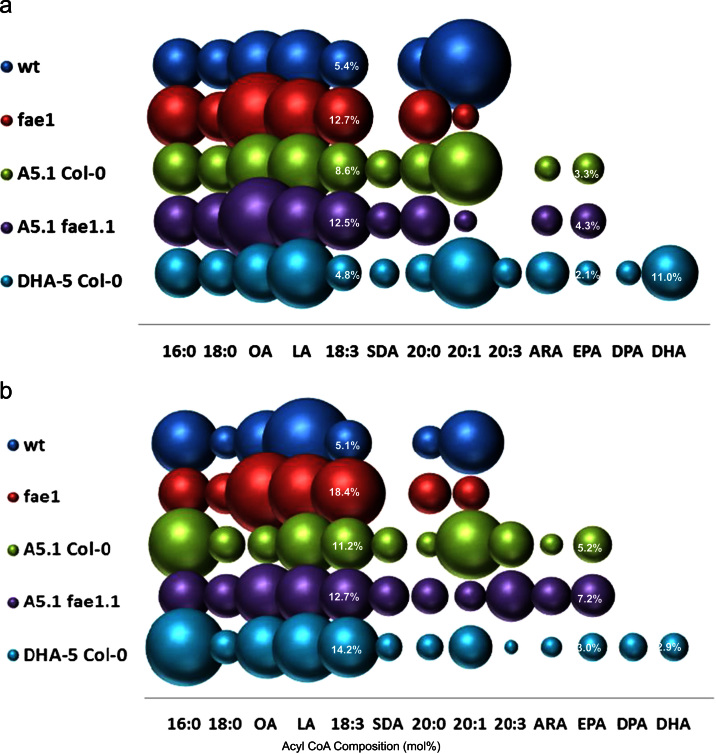
Graphical representation of the accumulation of acyl-CoAs in transgenic Arabidopsis plants containing constructs DHA-1 to DHA-5. The mean mol% of individual fatty acids (*Y*-axis) is directly proportional to the size of the sphere. Different constructs are shown on the *X* axis as indicated. For reference, the mol% accumulation of the target fatty acids EPA and DHA are given numerically within the relevant spheres, as is the mol% for the endogenous fatty acid ALA. A full description of the fatty acid composition of these lines is given in Supplementary Table 7. Lines are T2 generation, panel A is for seeds at mid-stage seed development (18 days after flowering), B is for mature seeds.

To further investigate the flux of non-native fatty acids between phospholipids and neutral lipids (Fig. 8), acyl-CoA profiling was carried out on both mature and developing *Arabidopsis* seeds of the same lines (Fig. 9; Supp. Table 7). Of particular note is the accumulation of DHA-CoA in the developing seeds (18 DAF) of line DHA-5; levels of this particular acyl-CoA are present at 11%, yet DHA-CoA declined markedly to 2.9% in mature seeds (Fig. 9; Supp. Table 7). Given that the only lipid species which accumulated DHA to relatively high levels was PE (9.5%; Table 2); these data suggest a number of scenarios. Firstly, that DHA-CoA is preferentially incorporated into PE, depleting this acyl-CoA species from the pool and reducing potential flux into other (more preferable) lipids such as TAG and/or DAG (Fig. 8). Incorporation of DHA into PE is most likely mediated by LPAAT-type activities (Stålberg et al., 2010), but their ability to recognise non-native *n*−3 LC-PUFAs as substrates has not previously been examined. Another scenario is that as the developing *Arabidopsis* seed progresses through development, significantly higher levels of other (endogenous) fatty acids enter the acyl-CoA pool, diluting the levels of DHA-CoA and other non-native fatty acids. However, EPA-CoA and DPA-CoA do not decrease in mature seeds compared with 18 days after flowering developing seeds (Fig. 9), making this seem less likely. Finally, it could be hypothesised that DHA-CoA is preferentially catabolised, though the basis for this remains obscure. Whatever the situation, it is clear that whilst DHA can accumulate to appreciable levels in the acyl-CoA pool of the developing seeds of *Arabidopsis* this is not reflected by a similar level of accumulation in neutral lipids, although it is equally clear that this non-native fatty acid is not actively excluded from TAG or DAG. In that respect, it seems most likely that DHA is directly incorporated into *Arabidopsis* lipids (both neutral and polar) via acyl-CoA-dependent pathways such as the Kennedy pathway, as opposed to remodelling and/or head-group exchange (Fig. 8) (Napier, 2007; Venegas-Calerón et al., 2010). This is supported by the very low levels of DHA in glycolipids (MGDG, DGDG; Table 4) which are synthesised from a DAG intermediate. Ultimately, these data indicate that it may be necessary to enhance the flux of DHA-CoA into target lipids, most likely through the co-expression of an appropriate acyltransferase such as DGAT2 (Li et al., 2012) to direct the incorporation of DHA into the sn-3 position of TAG.

## 4 Conclusions

In this study we have systematically evaluated the role of thirteen different genes in twelve different combinations and in two different host genetic backgrounds for their capacity to synthesise *n*−3 LC-PUFAs. To our knowledge, this is the largest systematic analysis of genetic factors contributing to the transgenic accumulation of these non-native fatty acids. All twelve combinations contained the same three “core” activities known to be required for EPA synthesis (Δ6-desaturase, Δ6-elongase, Δ5-desaturase; Fig. 1), with the optimal combination of different orthologs previously identified by us (Sayanova et al., 2011, 2012). Thus, these three genes formed the basis of a first iteration; which was then expanded six times to generate EPA and a further five times to direct the synthesis of DHA (Fig. 1A and B).

These analyses have identified a preferential combination of transgenes, and surprisingly, indicated that there was only modest benefit from the co-expression of additional activities. For example, the most sophisticated iterations of the EPA constructs (A6.1, A6.2), which contained three additional genes compared to the A3.1 primary construct, only showed a 1.2 to 1.25-fold increase in total seed EPA levels. Interestingly, some of the additional activities co-expressed with the three primary genes resulted in significant alteration of the endogenous composition of *Arabidopsis* seed fatty acids e.g. expression of the *P. frutescans* FAD3 desaturase significantly increased the total levels of ALA. However, these increased levels of ALA did not lead to higher EPA or flux through the transgene-derived pathway, emphasising the importance of acyl-exchange in the successful metabolic engineering of this pathway. In addition, we observed that the preferred substrate for the OtΔ6-desaturase was the *n*−6 LA, as opposed to *n*−3 ALA, indicating a potential disadvantage associated with this particular acyl-CoA-dependent desaturase.

A related situation was observed with our use of the *fae1-1* mutant background, which is blocked in the elongation of oleic acid to 20:1*n*−9; consequently the *fae1-1* mutant background accumulates higher levels of oleic acid and LA. In theory, these fatty acids should serve as substrates for either endogenous FAD2 and FAD3 desaturases or equivalent activities present in our A4-onwards iterations (Fig. 1). Interestingly, expression in the *fae1-1* background only yields minor increases in the accumulation of EPA, although it was notable that this did increase significantly in the T3 generation to 13% (A6.1/*fae1-1*) compared to the WT background (9.6%, A6.1/WT). However, even these boosts to the EPA level represent only a relatively small percentage of the fatty acids redirected from 20:1 synthesis as a consequence of the *fae1-1* mutation. In that respect it is very clear that a better understanding of the flux of fatty acids into lipids is still required, despite recent advances through the work of Bates and Browse (2011, 2012) and analogous studies in other plants (Alonso et al., 2011). Collectively, this means that adopting a rational approach to the manipulation of plant seed oil composition is still fraught with problems, not least of all when our understanding of these metabolic pathways in both host and donor organisms is partial (Radakovits et al., 2010; Ruiz-Lopez et al., 2012).

In conclusion, our study has identified an optimal set of genes with which to successfully direct the transgenic synthesis of EPA or DHA. Surprisingly, expression of additional activities to modulate substrates did not result in a stoichiometric increase in the levels of these target fatty acids. However, as graphically shown in Fig. 6, over the course of the iterations described in this study we have more than doubled the transgenic accumulation of EPA in *Arabidopsis*. Given the relatively modest levels (10–25% of total) of this fatty acid in *bona fide* fish oils, our data indicate the benefits of a systematic approach to the engineering of plant lipid metabolism.

## Figures and Tables

**Table 1:** Fatty acid composition (mol%) of neutral lipids isolated from seeds of wild type (WT; Col-0), *fae1-1* mutant (*fae1-1*) and transgenic *Arabidopsis* lines producing EPA and DHA.

	16:0	18:0	18:1	18:2	GLA	ALA	SDA	20:1	DGLA	ARA	ETA	EPA	DPA	DHA	Others
*Total Neutral lipids*															
WT (Col-0)	7.1	3.5	16.3	27.4	0.0	17.2	0.0	21.2	0.0	0.0	0.0	0.0	0.0	0.0	7.2
f*ae1-1*	9.2	4.2	27.7	38.2	0.3	18.3	0.0	0.6	0.0	0.0	0.0	0.0	0.0	0.0	1.5
A5 .1/Col-0	7.5	3.7	11.1	25.4	3.4	11.5	2.1	17.7	0.2	1.4	0.4	8.7	0.0	0.0	6.9
A5.1/*fae1-1*	10.0	4.7	18.3	31.5	4.4	13.6	2.3	1.6	0.2	1.6	0.2	8.5	0.0	0.0	3.1
DHA-5	9.0	4.7	10.7	28.2	1.4	13.0	0.6	14.7	0.8	1.6	1.2	3.7	0.8	2.5	7.1
TAG															
WT	8.2	3.5	16.4	27.3	0.0	17.4	0.0	20.3	0.0	0.0	0.0	0.0	0.0	0.0	6.9
*fae1-1*	10.0	4.0	27.0	37.5	0.9	18.3	0.0	0.9	0.0	0.0	0.0	0.0	0.0	0.0	1.5
A5.1/Col-0	8.8	3.7	11.2	26.0	3.4	11.8	2.0	16.4	0.1	1.3	0.4	8.2	0.0	0.0	6.6
A5.1/*fae1-1*	11.6	4.6	18.4	31.2	4.8	13.5	2.4	1.6	0.1	1.4	0.2	7.5	0.0	0.0	2.7
DHA-5	9.6	4.5	10.6	28.1	1.3	13.3	0.5	14.6	0.8	1.6	1.2	3.7	0.7	2.5	6.9

DAG															
WT	7.8	3.6	17.1	36.1	0.0	14.4	0.0	14.7	0.0	0.0	0.0	0.0	0.0	0.0	6.2
*fae1-1*	8.8	4.3	25.4	39.4	0.4	19.4	0.0	0.8	0.0	0.0	0.0	0.0	0.0	0.0	1.5
A5.1/Col-0	7.5	3.8	10.9	26.5	5.0	11.2	2.5	13.6	0.0	1.0	0.7	10.4	0.0	0.0	6.8
A5.1/*fae1-1*	8.3	4.4	15.5	32.7	4.7	15.0	2.8	1.4	0.1	1.1	0.3	10.3	0.0	0.0	3.4
DHA-5	8.9	4.0	9.8	28.1	2.7	11.9	1.0	10.7	0.7	1.3	2.4	4.9	2.7	4.3	6.6

FFA															
WT	10.1	6.6	17.3	25.4	0.0	13.1	0.0	19.4	0.0	0.0	0.0	0.0	0.0	0.0	8.0
*fae1-1*	15.0	13.6	23.4	27.6	0.4	13.7	0.0	1.0	0.0	0.0	0.0	0.0	0.0	0.0	5.3
A5.1/Col-0	11.2	8.5	12.4	20.1	3.6	9.5	2.0	16.6	0.0	1.0	0.3	6.3	0.0	0.0	8.5
A5.1/*fae1-1*	11.6	10.6	18.5	26.0	4.0	11.9	2.2	1.6	0.1	1.4	0.1	7.6	0.0	0.0	4.3
DHA-5	10.1	5.2	8.1	25.1	1.6	14.2	0.7	13.9	0.9	1.8	1.5	4.1	1.1	4.0	7.6

**Table 2:** Fatty acid composition (mol%) of polar lipids isolated from seeds of wild type (WT; Col-0), *fae1-1* mutant (*fae1-1*) and transgenic *Arabidopsis* lines producing EPA and DHA.

	16:0	18:0	18:1	18:2	GLA	ALA	SDA	20:1	DGLA	ARA	ETA	EPA	DPA	DHA	Others
*Total Polar Lipids*															
WT (Col-0)	11.0	2.7	14.5	49.2	0.0	12.3	0.0	5.0	0.0	0.0	0.0	0.0	0.0	0.0	5.3
*fae1-1*	12.4	2.0	16.8	55.2	0.0	10.9	0.0	0.5	0.0	0.0	0.0	0.0	0.0	0.0	2.1
A5.1/Col-0	15.0	3.5	7.2	24.8	11.5	9.3	5.6	4.6	0.0	0.0	1.8	11.5	0.0	0.0	5.1
A5.1/*fae1-1*	14.1	3.0	8.9	35.5	9.3	14.4	4.9	0.1	0.0	0.0	0.3	7.6	0.0	0.0	2.0
DHA-5	15.9	3.4	6.2	27.9	5.0	9.0	1.9	3.1	0.3	0.1	3.8	7.3	4.7	6.8	4.5

*PC*															
WT	8.8	7.7	15.6	41.1	0.0	11.3	0.0	6.7	0.0	0.0	0.0	0.0	0.0	0.0	8.7
*fae1-1*	9.0	1.7	19.2	56.0	0.2	11.6	0.0	0.3	0.0	0.0	0.0	0.0	0.0	0.0	2.1
A5.1/Col-0	12.1	3.7	7.9	24.5	12.1	9.1	6.4	5.5	0.1	0.1	1.8	10.8	0.0	0.0	5.9
A5.1/*fae1-1*	11.6	3.1	9.4	36.2	9.9	14.9	5.6	0.2	0.0	0.0	0.4	6.2	0.0	0.0	2.5
DHA-5	11.5	3.9	7.3	27.8	5.0	10.0	2.0	5.8	0.5	0.4	3.8	5.9	4.4	5.5	6.0

*PE*															
WT	13.3	2.5	12.0	53.4	0.0	10.5	0.0	2.9	0.0	0.0	0.0	0.0	0.0	0.0	5.3
*fae1-1*	15.8	3.4	13.3	56.8	0.3	8.8	0.0	0.0	0.0	0.0	0.0	0.0	0.0	0.0	1.7
A5.1/Col-0	18.2	3.7	7.6	27.0	11.9	8.6	4.7	3.0	0.0	0.1	1.4	10.6	0.0	0.0	3.4
A5.1/*fae1-1*	17.0	2.9	9.3	35.1	9.5	12.6	4.0	0.2	0.0	0.0	0.2	7.8	0.0	0.0	1.5
DHA-5	18.8	3.6	6.4	27.0	4.0	8.0	1.2	2.1	0.1	0.0	3.4	7.6	4.9	9.5	3.4

*PI+PS*															
WT	24.8	5.6	8.8	40.4	0.0	12.9	0.0	2.0	0.0	0.0	0.0	0.0	0.0	0.0	5.6
*fae1-1*	33.7	4.0	8.8	39.3	0.0	10.0	0.0	1.2	0.0	0.0	0.0	0.0	0.0	0.0	3.1
A5.1/Col-0	36.6	4.1	4.5	22.7	5.9	8.0	2.2	1.5	0.0	0.1	0.3	9.8	0.0	0.0	4.4
A5.1/*fae1-1*	36.6	3.7	5.1	26.4	4.7	9.8	1.7	0.8	0.0	0.0	0.0	8.3	0.0	0.0	3.1
DHA-5	35.1	4.7	3.0	24.7	2.1	8.0	0.6	1.2	0.0	0.0	1.5	11.7	1.5	1.7	3.9

**Table 3:** Positional analysis of phospholipids isolated from seeds of transgenic *Arabidopsis* lines accumulating DHA.

DHA-5	16:0	18:0	18:1	18:2	GLA	ALA	SDA	20:1	DGLA	ARA	ETA	EPA	DPA	DHA	Others
PC (sn-1)	16.7	5.9	10.2	27.9	2.7	12.5	1.2	6.9	0.3	0.2	2.7	3.0	0.6	2.9	6.2
PC (sn-2)	5.5	1.9	3.5	28.5	9.3	6.1	3.3	1.3	0.6	0.2	6.2	9.8	9.6	9.0	5.1

**Table 4:** Fatty acid composition (mol%) of total glycolipids isolated from seeds of wild type (WT; Col-0), *fae1-1* mutant (*fae1-1*) and transgenic *Arabidopsis* lines producing EPA and DHA.

	16:0	18:0	18:1	18:2	GLA	ALA	SDA	20:1	DGLA	ARA	ETA	EPA	DPA	DHA	Others
*Glycolipids*															
WT (Col-0)	21.1	14.9	7.9	14.6	0.0	22.7	0.0	5.3	0.0	0.0	0.0	0.0	0.0	0.0	13.6
*fae1-1*	22.8	7.8	15.1	14.9	0.0	23.6	0.0	1.1	0.0	0.0	0.0	0.0	0.0	0.0	14.7
A5.1/Col-0	20.3	6.8	11.7	14.7	2.8	16.4	1.6	7.8	0.0	0.4	0.1	3.8	0.0	0.0	13.5
A5.1/*fae1-1*	18.9	6.1	14.2	19.0	2.9	18.7	1.6	2.1	0.0	0.5	0.0	4.7	0.0	0.0	11.2
DHA-5	22.7	10.4	13.7	13.2	1.2	10.5	0.3	12.6	0.2	0.4	0.4	1.1	0.3	1.2	12.0

## Footnotes

Appendix A Supplementary data associated with this article can be found in the online version at doi:10.1016/j.ymben.2013.03.001.
