# Supplementary material for: Reconstitution of EPA and DHA biosynthesis in Arabidopsis: Iterative metabolic engineering for the synthesis of n−3 LC-PUFAs in transgenic plants
Source: Metab Eng. 2013 May;17:30–41. doi: 10.1016/j.ymben.2013.03.001 (PMC3650579; doi:10.1016/j.ymben.2013.03.001)
Supplement: Supplementary file 1 — Supplementary data [file mmc1.docx]

**Supplementary Tables**

| Fatty acid | WT | A3.1 | A4.1 | A4.2 | A4.3 |
| --- | --- | --- | --- | --- | --- |
|  |  |  |  |  |  |
| 16:0 | 6.2 ± 0.2 | 10.4 ± 0.8 | 9.4 ± 0.4 | 9.3 ± 0.4 | 6.6 ± 0.4 |
| 18:0 | 3.3 ± 0.2 | 3.8 ± 0.3 | 3.6 ± 0.1 | 3.8 ± 0.1 | 3.3 ± 0.2 |
| 18:1n-9 | 14.5 ± 0.5 | 10.6 ± 1.4 | 14.0 ± 1.1 | 15.2 ± 1.8 | 13.4 ± 1.2 |
| 18:2n-6 (LA) | 27.9 ± 0.6 | 26.6 ± 1.9 | 22.4 ± 1.1 | 13.0 ± 3.9 | 21.1 ± 1.7 |
| 18:3n-6 (GLA) | - | 1.7 ± 1.2  (0.4-3.9) | 1.4 ± 0.5  (0.6-2.2) | 1.0 ± 0.7  (0.2-2.4) | 2.3 ± 1.0  (0.6–4.7) |
| 18:3n-3 (ALA) | 15.7 ± 0.4 | 13.6 ± 0.7 | 12.7 ± 0.5 | 21.5 ± 2.6 | 10.7 ± 0.9 |
| 18:4n-3 (SDA) | - | 1.2 ± 0.7  (0.4-2.1) | 1.9 ± 0.8  (0.9-2.9) | 2.1 ± 1.1  (0.7-4.6) | 1.0 ± 0.4  (0.5–2.2) |
| 20:0 | 2.5 ± 0.1 | 2.0 ± 0.5 | 1.6 ± 0.1 | 1.7 ± 0.2 | 2.0 ± 0.2 |
| 20:1n-9 | 20.5 ± 0.2 | 12.0 ± 2.1 | 14.7 ± 0.8 | 14.9 ± 0.6 | 16.2 ± 1.2 |
| 20:3n-6 (DGLA) | - | 0.5 ± 0.3 | 0.4 ± 0.1 | 0.2 ± 0.2 | 0.7 ± 0.3 |
| 20:4n-6 (ARA) | - | 2.2 ± 2.0  (0.4 -6.4) | 4.6 ± 0.6  (4.0–5.8) | 1.5 ± 0.5  (0.8-2.5) | 3.3 ± 0.9  (1.8-4.7) |
| 20:4n-3 (ETA) | - | 1.4 ± 0.4 | 0.7 ± 0.1 | 1.1 ± 0.5 | 0.4 ± 0.3 |
| 20:5n-3 (EPA) | - | 5.7 ± 1.2  (3.5–7.5) | 4.3 ± 0.5  (3.4-5.3) | 5.8 ± 1.0  (4.4-7.4) | 5.7 ± 2.2  (2.9-10.4) |
| Others | 9.4 ± 0.2 | 8.3 ± 0.9 | 8.3 ± 0.4 | 9.0 ± 1.0 | 13.4 ± 1.3 |
| New n-6 | - | 4.4 ± 2.3 | 6.3 ± 0.7 | 2.7 ± 1.0 | 6.3 ± 1.2 |
| New n-3 | - | 8.9 ± 1.8 | 6.9 ± 1.3 | 8.9 ± 1.2 | 7.1 ± 2.1 |

**Supplementary Table 1.**

Total fatty acids composition of T2 seeds from transgenic Arabidopsis lines, expressing triple- gene core and four-gene construct. Highest and lowest levels observed are given in brackets for non-native fatty acids. See Supplementary Figures for graphical representation of these data.

|  |  | 16:0 | 16:1 | 18:0 | 18:1 | 18:2ω6 | 18:3ω6 | 18:3ω3 | 18:4ω3 | 20:3ω6 | 20:4ω6 | 20:4ω3 | 20:5ω3 |
| --- | --- | --- | --- | --- | --- | --- | --- | --- | --- | --- | --- | --- | --- |
|  |  |  |  |  | OA | LA | GLA | ALA | SDA | DGLA | ARA | ETA | EPA |
|  | +Glu | 25.7 | 43.3 | 5.4 | 25.7 | - | - | - | - | - | - | - | - |
|  | +Gal | 24.1 | 43.0 | 5.5 | 27.3 | - | - | - | - | - | - | - | - |
| +LA | +Gal | 26.8 | 34.1 | 5.6 | 21.0 | 12.4 | - | 0.2 | - | - | - | - | - |
| +GLA | +Gal | 26.3 | 8.1 | 5.8 | 5.2 | - | 54.2 | - | 0.3 | - | - | - | - |
| +DGLA | +Gal | 27.2 | 37.7 | 5.8 | 22.3 | - | - | - | - | 6.3 | - | 0.6 | - |
| +ARA | +Gal | 28.0 | 36.9 | 6.1 | 21.5 | - | - | - | - | - | 5.7 | - | 1.8 |

**Supplementary Table 2**.

Fatty acid profiles in lipids of yeast expressing a new C20-specific ω3-desaturase from *Hyaloperonospora parasitica*, Hp-ω3, in the presence of different supplemented fatty acids. The conversion of ARA to EPA confirms that this enzyme acts as C20-specific ω3-desaturase

**Supplementary Table 3.**

Total fatty acids composition of T2 seeds from transgenic Arabidopsis lines, expressing 5- and 6-gene constructs. Highest and lowest levels observed are given in brackets for non-native fatty acids. See Supplementary Figures for graphical representation of these data.

| Fatty acid | WT | A5.1 | A6.1 | A6.2 |
| --- | --- | --- | --- | --- |
|  |  |  |  |  |
| 16:0 | 6.2 ± 0.2 | 5.1 ± 1.2 | 3. 8 ± 0.4 | 4.5 ± 0.6 |
| 18:0 | 3.3 ± 0.2 | 3.7 ± 0.2 | 3.6 ± 0.2 | 3.5 ± 0.2 |
| 18:1n-9 | 14.5 ± 0.5 | 12.4 ± 1.1 | 11.1 ± 1.3 | 11.1 ± 1.0 |
| 18:2n-6 | 27.9 ± 0.6 | 23.8 ± 2.4 | 22.0 ± 1.6 | 15.5 ± 2.3 |
| 18:3n-6 | - | 1.9 ± 1.5  (0.2 –6.1) | 1.1 ± 0.8  (0.3–3.7) | 0.7 ± 0.6  (0.2-3.1) |
| 18:3n-3 | 15.7 ± 0.4 | 11.8 ± 1.1 | 12.3 ± 0.8) | 18.9 ± 2.1 |
| 18:4n-3 | - | 1.4 ± 1.0  (0.1–3.7) | 1.2 ± 0.9  (0.2-3.9) | 1.9 ± 1.5  (0.5-6.9) |
| 20:0 | 2.5 ± 0.1 | 2.7 ± 0.3 | 3.0 ± 0.2 | 3.0 ± 0.3 |
| 20:1n-9 | 20.5 ± 0.2 | 21.7 ± 2.3 | 24.1 ± 1.2 | 23.6 ± 1.9 |
| 20:3n-6 | - | 0.5 ± 0.3 | 0.4 ± 0.3 | 0.3 ± 0.2 |
| 20:4n-6 | - | 1.0 ± 0.3  (0.4–1.8) | 1.2 ± 0.4  (0.7–2.8) | 0.7 ± 0.3  (0.3-1.4) |
| 20:4n-3 | - | 0.9 ± 0.4 | 0.8 ± 0.4 | 0.9 ± 0.5 |
| 20:5n-3 | - | 5.8 ± 1.4  (4.3–10.5) | 6.9 ± 1.7  (4.1–11.7) | 7.1 ± 2.1  (3.7–12.4) |
| Others | 9.4 ± 0.2 | 7.2 ± 1.0 | 8.6 ± 1.0 | 8.4 ± 1.2 |
| New n-6 | - | 3.2 ± 1.1 | 2.7 ± 0.7 | 1.7 ± 0.7 |
| New n-3 | - | 8.0 ± 1.7 | 8.9 ± 1.8 | 9.9 ± 1.8 |

**Supplementary Table 4**.

Total fatty acids composition of T2 seeds from transgenic Arabidopsis lines in *fae1-1* mutant. Highest and lowest levels observed are given in brackets for non-native fatty acids. See Supplementary Figures for graphical representation of these data.

|  | *fae1-1* | A5.1 *fae1-1* | A6.1 *fae1-1* |
| --- | --- | --- | --- |
| Fatty acid |  |  |  |
| 16:0 | 8.9 ± 0.5 | 6.0 ± 0.6 | 7.6 ± 1.1 |
| 18:0 | 4.5 ± 0.1 | 4.8 ± 0.4 | 4.7 ± 0.3 |
| 18:1n-9 | 27.2 ± 1.5 | 21.0 ± 2.2 | 21.6 ± 1.5 |
| 18:2n-6 | 37.2 ± 1.1 | 30.4 ± 1.5 | 31.6 ± 1.5 |
| 18:3n-6 | - | 2.4 ± 1.5  (0.9–5.8) | 1.7 ± 1.3  (0.4–4.7) |
| 18:3n-3 | 17.3 ± 0.1 | 13.9 ± 0.7 | 14.3 ± 0.8 |
| 18:4n-3 | - | 1.9 ± 1.1  (0.5–4.1) | 1.4 ± 0.8  (0.5–3.2) |
| 20:0 | 0.9 ± 0.1 | 1.5 ± 0.1 | 1.1 ± 0.1 |
| 20:1n-9 | 0.4 ± 0.1 | 1.3 ± 0.6 | 1.1 ± 0.3 |
| 20:3n-6 | - | 0.6 ± 0.4 | 0.5 ± 0.3 |
| 20:4n-6 | - | 1.7 ± 0.8  (0.7–3.8) | 1.6 ± 0.4  (0.8–2.1) |
| 20:4n-3 | - | 1.0 ± 0.4 | 1.0 ± 0.4 |
| 20:5n-3 | - | 8.3 ± 2.0  (5.3–13.2) | 7.3 ± 1.7  (4.9–10.6) |
| Others | 3.6 ± 0.1 | 5.2 ± 1.3 | 4.5 ± 0.9 |
| New n-6 | - | 4.7 ± 1.5 | 3.9 ± 1.0 |
| New n-3 | - | 11.3 ± 2.2 | 9.6 ± 1.8 |

**Supplementary Table 5**.

Total fatty acids composition of the T3 seeds from selected Arabidopsis lines. Highest and lowest levels observed are given in brackets for non-native fatty acids.

|  | WT  Col-0 | *fae1-1* | A4.3  Col-0 | A5.1  Col-0 | | A5.1 *fae1-1* | | A6.1  Col-0 | A6.1  *fae1-1* |
| --- | --- | --- | --- | --- | --- | --- | --- | --- | --- |
| *Fatty acid* |  |  |  | |  |  |  | |  |
| *16:0* | 6.2 ± 0.2 | 8.9 ± 0.5 | 10.2 ± 0.7 | | 6.3 ± 1.2 | 7.1 ± 0.9 | 4.5 ± 0.4 | | 7.4 ± 1.0 |
| *18:0* | 3.3 ± 0.2 | 4.5 ± 0.1 | 3.8 ± 0.3 | | 3.9 ± 0.1 | 5.2 ± 0.2 | 3.6 ± 0.2 | | 4.8 ± 0.3 |
| *18:1n-9* | 14.5 ± 0.5 | 27.2 ± 1.5 | 11.5 ± 0.9 | | 8.1 ± 0.7 | 16.2 ± 1.1 | 7.7 ± 0.8 | | 12.3 ± 2.4 |
| *18:2n-6* | 27.9 ± 0.6 | 37.2 ± 1.1 | 22.3 ± 1.7 | | 23.9 ± 0.9 | 30.4 ± 1.0 | 21.6 ± 1.5 | | 28.1 ± 2.8 |
| *18:3n-6* | - | - | 1.9 ± 0.7  (0.9-3.2) | | 2.0 ± 1.2  (0.7–5.0) | 1.8 ± 0.8  (1.0–3.1) | 1.8 ± 1.0  (0.5–4.7) | | 1.9 ± 1.1  (1.0–4.0) |
| *18:3n-3* | 15.7 ± 0.4 | 17.3 ± 0.1 | 11.5 ± 1.1 | | 11.3 ± 0.7 | 12.3 ± 0.4 | 11.5 ± 0.7 | | 12.5 ± 0.7 |
| *18:4n-3* | - | - | 0.8 ± 0.3 | | 0.8 ± 0.3 | 0.7 ± 0.2 | 1.1 ± 0.4 | | 1.1 ± 0.2 |
| *20:0* | 2.5 ± 0.1 | 0.9 ± 0.1 | 1.7 ± 0.2 | | 2.7 ± 0.2 | 1.5 ± 0.2 | 3.0 ± 0.2 | | 1.3 ± 0.2 |
| *20:1n-9* | 20.5 ± 0.2 | 0.4 ± 0.1 | 12.0 ± 1.3 | | 16.0 ± 1.5 | 0.3 ± 0.1 | 17.0 ± 1.6 | | 0.4 ± 0.3 |
| *20:3n-6* | - | - | 1.4 ± 0.6 | | 1.7 ± 0.5 | 1.1 ± 0.5 | 2.1 ± 0.5 | | 1.5 ± 0.3 |
| *20:4n-6* | - | - | 3.4 ± 0.4  (2.6-4.0) | | 3.2 ± 0.4  (0.4-3.8) | 3.4 ± 0.6  (2.2-4.6) | 3.3 ± 0.5  (2.4-4.0) | | 3.2 ± 0.5  (2.1-4.4) |
| *20:4n-3* | - | - | 1.0 ± 0.4 | | 1.5 ± 0.2 | 1.1 ± 0.2 | 1.8 ± 0.4 | | 0.9 ± 0.3 |
| *20:5n-3* | - | - | 7.3 ± 1.4  (5.8–10.0) | | 8.9 ± 1.2  (7.0–11.9) | 10.2 ± 1.3  (7.9–12.2) | 9.6 ± 2.0  (6.1–13.0) | | 13.2 ± 2.2  (9.4–16.7) |
| *Others* | 9.4 ± 0.2 | 3.6 ± 0.1 | 12.0 ± 1.3 | | 10.5 ± 0.2 | 8.7 ± 0.5 | 11.4 ± 1.9 | | 11.4 ± 2.2 |
| *New n-6* | - | - | 6.7 ± 1.1 | | 6.8 ± 0.7 | 6.2 ± 0.5 | 7.2 ± 0.6 | | 6.6 ± 0.7 |
| *New n-3* | - | - | 9.1 ± 1.4 | | 10.4 ± 1.2 | 11.1± 1.4 | 11.1 ± 1.9 | | 14.3 ± 2.2 |
|  |  |  |  | |  |  |  | |  |

**Supplementary Table 6**.

Total fatty acids composition of T2 seeds from transgenic Arabidopsis lines, expressing DHA- constructs. Highest and lowest levels observed are given in brackets for non-native fatty acids. See Supplementary Figures for graphical representation of these data.

|  | DHA-1 DHA-2 | | DHA-3 | DHA-4 DHA-5 | |
| --- | --- | --- | --- | --- | --- |
|  | T2  (n=8) | T2  (n=13) | T2  (n=13) | T2  (n=20) | T2  (n=24) |
|  |  |  |  |  |  |
| 16:0 | 6.9 ± 0.2 | 9.6 ± 0.5 | 8.9 ± 0.6 | 6.4 ± 0.5 | 7.2 ± 0.9 |
| 18:0 | 3.4 ± 0.2 | 3.7 ± 0.2 | 3.8 ± 0.3 | 3.6 ± 0.2 | 3.4 ± 0.2 |
| 18:1n-9 | 10.8 ± 0.9 | 11.0 ± 1.4 | 10.5 ± 1.6 | 9.4 ± 1.9 | 9.5 ± 1.5 |
| 18:2n-6 | 21.1 ± 1.4 | 28.4 ± 1.4 | 27.7 ± 2.0 | 24.0 ± 1.7 | 24.5 ± 1.9 |
| 18:3n-6 | 1.5 ± 0.8  (0.3-2.5) | 2.1 ± 1.5  (0.4-5.0) | 1.7 ± 1.6  (0.3-6.1) | 1.8 ± 0.9  (0.5-3.5) | 2.8 ± 1.5  (0.6-5.7) |
| 18:3n-3 | 11.2 ± 1.1 | 12.3 ± 0.8 | 12.4 ± 1.1 | 12.4 ± 1.0 | 12.4 ± 1.1 |
| 18:4n-6 | 0.7 ± 0.3 | 2.5 ± 0.4 | 2.2 ± 0.3 | 1.0 ± 0.6 | 1.6 ± 0.9 |
| 20:1n-9 | 14.5 ± 1.1 | 14.5 ± 0.7 | 14.0 ± 4.2 | 16.0 ± 1.5 | 15.2 ± 1.3 |
| 20:3n-6 | 1.4 ± 0.7 | 0.4 ± 0.2 | 0.5 ± 0.1 | 0.5 ± 0.3 | 0.6 ± 0.2 |
| 20:4n-6 | 3.1 ± 1.0  (1.9-4.5) | 0.8 ± 0.3  (0.2-1.2) | 1.2 ± 0.2  (0.9-1.5) | 1.1 ± 0.3  (0.4-1.6) | 1.0 ± 0.4  (0.6-2.6) |
| 20:5n-3 | 4.8 ± 0.9  (3.2-5.7) | 3.5 ± 1.8  (1.0-6.0) | 5.9 ± 1.6  (2.2-8.8) | 3.2 ± 1.1  (1.1-5.4) | 3.4 ± 1.4  (1.9-7.9) |
| 22:5n-3 | 1.1 ± 0.2 | 0.7 ± 0.3 | 0.3 ± 0.3 | 2.7 ± 0.8 | 1.1 ± 0.5 |
| 22:6n-3 | 1.3±0.4  (0.9-2.0) | 0.6 ± 0.3  (0.3-1.1) | 0.3 ± 0.2  (0.2-0.8) | 2.0 ± 0.7  (1.1-3.8) | 2.5 ± 1.0  (0.9-4.7) |
| Others | 18.2 ± 1.3 | 10.1 ± 1.1 | 10.8 ± 1.0 | 16.0 ± 1.7 | 14.7 ± 2.0 |
| New n-6 | 6.1 ± 2.1 | 3.3 ± 1.3 | 3.3 ± 1.5 | 3.4 ± 0.9 | 4.4 ± 1.3 |
| New n-3 | 7.9 ± 1.3 | 7.2 ± 2.1 | 8.6 ± 1.7 | 8.8 ± 2.8 | 8.7 ± 2.4 |

**Supplementary Table 7**

Acyl CoA composition (mol%) isolated from seeds of wild type (WT; Col-0), *fae1-1* mutant (*fae1-1*) and transgenic Arabidopsis lines producing EPA and DHA. Acyl-CoA profiling does not resolve regio-isomers of 18:3. DAF = days after flowering (i.e. developing seeds)

|  | WT  Col-0 | *fae1-1* | A5.1  Col-0 | A5.1  *fae1-1* | DHA-5  Col-0 |
| --- | --- | --- | --- | --- | --- |
|  |  |  |  |  |  |
| *18DAF* |  |  |  |  |  |
| 16:0 | 8.8 | 14.4 | 9.3 | 8.1 | 8.3 |
| 18:0 | 8.5 | 8.4 | 5.9 | 11.2 | 7.1 |
| 18:1 | 16.5 | 25.3 | 17.6 | 18.4 | 12.1 |
| 18:2 | 7.4 | 16.1 | 16.1 | 16.7 | 17.6 |
| 18:3 | 5.4 | 12.7 | 8.6 | 12.3 | 4.8 |
| SDA | 0.0 | 0.0 | 5.0 | 4.5 | 3.0 |
| 20:0 | 8.9 | 6.7 | 8.9 | 4.7 | 4.9 |
| 20:1 | 29.0 | 2.4 | 19.0 | 1.7 | 18.1 |
| DGLA | 0.0 | 0.0 | 0.0 | 0.0 | 3.0 |
| ARA | 0.0 | 0.0 | 2.2 | 3.2 | 6.3 |
| EPA | 0.0 | 0.0 | 3.3 | 4.3 | 2.1 |
| DPA | 0.0 | 0.0 | 0.0 | 0.0 | 2.1 |
| DHA | 0.0 | 0.0 | 0.0 | 0.0 | 11.0 |
|  |  |  |  |  |  |
| *Mature* |  |  |  |  |  |
| 16:0 | 16.5 | 10.0 | 20.9 | 10.4 | 23.8 |
| 18:0 | 4.4 | 6.8 | 5.3 | 7.6 | 4.5 |
| 18:1 | 12.6 | 26.9 | 5.8 | 16.2 | 15.5 |
| 18:2 | 31.2 | 16.1 | 15.1 | 15.3 | 18.5 |
| 18:3 | 5.1 | 18.4 | 11.2 | 12.7 | 14.2 |
| SDA | 0.0 | 0.0 | 4.9 | 6.4 | 2.8 |
| 20:0 | 2.3 | 6.7 | 2.5 | 5.0 | 2.5 |
| 20:1 | 15.9 | 4.7 | 18.9 | 3.6 | 7.0 |
| DGLA | 0.0 | 0.0 | 8.2 | 10.3 | 0.8 |
| ARA | 0.0 | 0.0 | 1.8 | 5.8 | 1.5 |
| EPA | 0.0 | 0.0 | 5.2 | 7.2 | 3.0 |
| DPA | 0.0 | 0.0 | 0.0 | 0.0 | 3.2 |
| DHA | 0.0 | 0.0 | 0.0 | 0.0 | 2.9 |
